# Extracellular vesicles: A dive into their role in the tumor microenvironment and cancer progression

**DOI:** 10.3389/fcell.2023.1154576

**Published:** 2023-03-21

**Authors:** Kassandra Lopez, Seigmund Wai Tsuen Lai, Edwin De Jesus Lopez Gonzalez, Raúl G. Dávila, Sarah C. Shuck

**Affiliations:** Department of Diabetes and Cancer Metabolism, Arthur Riggs Diabetes and Metabolism Research Institute, City of Hope Comprehensive Cancer Center, Duarte, CA, United States

**Keywords:** extracellular vesicles, cargo transport, extracellular signaling, cancer, extracellular matrix, tumor microenvironment, cancer-derived extracellular vesicles

## Abstract

Extracellular vesicles (EVs) encompass a diverse set of membrane-derived particles released from cells and are found in numerous biological matrices and the extracellular space. Specific classes of EVs include apoptotic bodies, exosomes, and microvesicles, which vary in their size, origin, membrane protein expression, and interior cargo. EVs provide a mechanism for shuttling cargo between cells, which can influence cell physiology by transporting proteins, DNA, and RNA. EVs are an abundant component of the tumor microenvironment (TME) and are proposed to drive tumor growth and progression by communicating between fibroblasts, macrophages, and tumor cells in the TME. The cargo, source, and type of EV influences the pro- or anti-tumoral role of these molecules. Therefore, robust EV isolation and characterization techniques are required to ensure accurate elucidation of their association with disease. Here, we summarize different EV subclasses, methods for EV isolation and characterization, and a selection of current clinical trials studying EVs. We also review key studies exploring the role and impact of EVs in the TME, including how EVs mediate intercellular communication, drive cancer progression, and remodel the TME.

## Introduction

**FIGURE 1:**
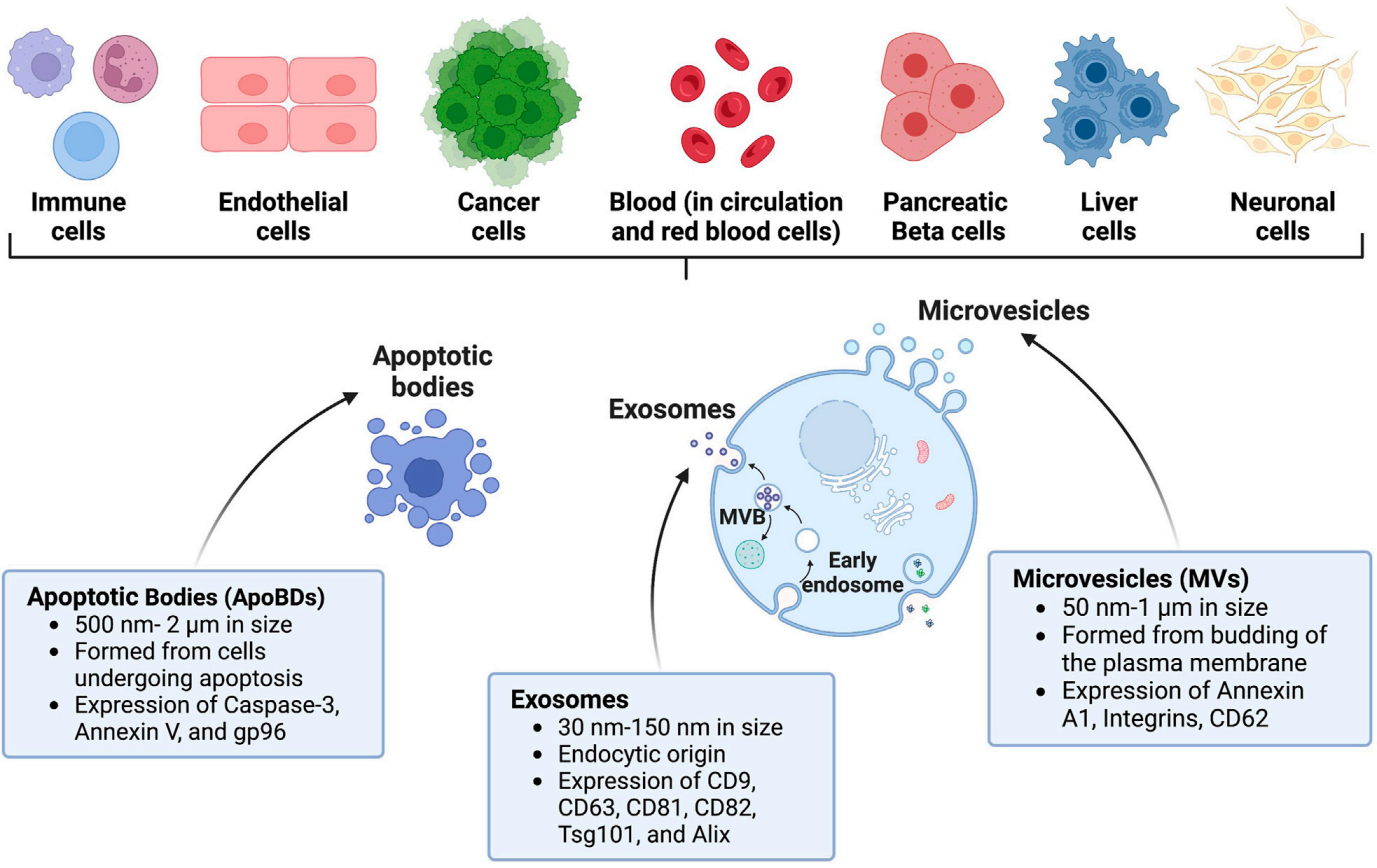
Characteristics of EVs and their origin. EVs can be shed by several diverse cell types throughout the body. Prominent examples of EVs include apoptotic bodies (ApoBDs), exosomes, and microvesicles (MVs). Created with BioRender.com.

Extracellular vesicles (EVs) are a heterogeneous group of non-replicating vesicles with a lipid bilayer that are secreted by cells into the extracellular space (Théry et al., 2018). Dr. D.W. Fawcett first observed these “virus-like particles” in 1956 and was among the first to define what we now know as EVs (Fawcett, 1956). Shortly after in 1967, Dr. Peter Wolf likened them to “platelet dust” (Wolf, 1967). Since their initial discovery, there has been a significant increase in studies investigating EVs, with the largest spike occurring in the past decade. A PubMed search of “extracellular vesicles” identified more than 12,000 manuscripts studying EVs published in 2021–2022. EVs are produced from almost all cell types and organisms, including humans and mice, and are proposed to act as mediators of intercellular communication by shuttling cargo between cells (Yoon et al., 2014). Three main subtypes of EVs have been described: apoptotic bodies (ApoBDs), microvesicles (MVs), and exosomes, which differ based on biogenesis, release pathway, size, cargo content, and membrane protein expression (Doyle and Wang, 2019). These differences are described in the EV subclassification section below and in Figure 1.

EVs are found in various biological matrices including whole blood, plasma, serum, saliva, urine, sweat, cerebrospinal fluid, and breast milk (Monguió-Tortajada et al., 2019). EVs have emerged as a source of potential biomarkers and as drivers of diseases including neurological diseases, diabetes, and cancer (Shetty and Upadhya, 2021). The diverse range of EV cargo includes various species of DNA, RNA, proteins, and lipids that reflect the physical state of the originating cell (Malkin and Bratman, 2020; Clos-Sansalvador et al., 2022). This cargo, therefore, can transfer physiological information from the originating cell to the recipient cell, serving to propagate the disease state and act as a potential biomarker of disease. Recent studies have focused on the role of EVs in regulating tumor cell growth and survival within the tumor microenvironment (TME). The TME consists of a diverse range of cellular and non-cellular components, including EVs. EV cargo can be transferred between cells in the TME such as fibroblasts and macrophages and cancer cells, impacting tumor growth (Cavallari et al., 2020). An emerging concept of “immunogenic stress” including autography (Guo et al., 2017), endoplasmic reticulum (ER) stress (Collett et al., 2018), and DNA damage (Lespagnol et al., 2008) has been implicated in changing the composition of RNAs, proteins, and lipids in EVs that mediate cellular cross talk within the TME. The heterogeneous combination of immune cells, resistant and infiltrating host cells, secreted factors, and extracellular matrix (ECM) components within the TME form either an anti-tumor immune competent microenvironment or pro-tumor immunosuppressive microenvironment (Anderson and Simon, 2020). In addition to the crosstalk between cancer and immune cells, exosomes and ApoBDs facilitate inter-and intra-tumoral communication. Tumor-derived EVs impact the TME and play a significant role in cancer progression. Tumor cells release more than 10^4^ EVs/day, as determined by using NanoSight LM10 nanoparticle analysis (Balaj et al., 2011). Medulloblastoma cells released 13,400-25,300 EVs per cell per 48 h, while normal fibroblast cells released 3,800-6,200 per cell per 48 h (Balaj et al., 2011). EVs have been isolated and studied from various solid tumors, including pancreatic (Nigri et al., 2022), lung (Hasan et al., 2022), breast (Rontogianni et al., 2019), and brain (Lane et al., 2019). These EVs can be detected in plasma from patients with breast, ovarian, prostate, hepatic, gastric, colon, and pancreatic cancers, which had elevated exosome levels compared to healthy subjects (Huang et al., 2019). This review will focus on EVs found within the TME, delve into their role in cancer progression, and describe potential applications of EVs in cancer therapeutics and diagnostics.

### EV subclassification

#### Exosomes

Exosomes are the smallest classified subtype of EVs ranging from 30–150 nm in size (Gurung et al., 2021). Exosomes were defined in the early 1980s by two seminal papers from Drs. Johnstone and Stahl (Harding et al., 1983; Pan and Johnstone, 1983). Johnstone coined the term exosome to describe EVs of endosomal origin and although it is widely accepted, it is being replaced by small extracellular vesicles (sEVs) due to difficulties in separating the heterogenous population of EVs (Johnstone et al., 1987; Willms et al., 2018). Exosomes are generated through the endosomal pathway after fusion of multivesicular bodies with the plasma membrane (Tkach and Théry, 2016; Hessvik and Llorente, 2018). One of the characteristics of exosomes are their enrichment with the following tetraspanins: CD9, CD63, CD81, CD82, heat shock proteins HSP70 and HSP90, Alix, and TSG101, which are involved in their release, and Annexins and Rab that play a role in membrane transport and fusion (Vlassov et al., 2012). Exosomes were initially proposed to function as cellular waste disposal or as a byproduct of homeostasis. However, the identification of their protein, lipid, and nucleic acid cargo has highlighted their importance in disease progression and as a diagnostic tool, as exosome cargo may provide information on which cell type it originated from, and if the cell is undergoing physiological changes such as cell stress, differentiation, and replication. Their small size allows exosomes to cross the blood-brain barrier (BBB) in a bidirectional manner; therefore, exosomes may have potential utility as drug delivery tools for neurological inflammatory and degenerative disorders (Heidarzadeh et al., 2021). Due to the endocytic origin of exosomes, they are commonly enriched in endosome-associated proteins including Rab, GTPases, soluble *N*-ethylmaleimide–sensitive factor attachment protein receptor (SNAREs), annexins, and flotillin, which play a role in the origin and biogenesis of exosomes (Kalluri and LeBleu, 2020).

#### Microvesicles (MVs)

MVs were first characterized in 1971 by Schrier et al. *via* sucrose gradient centrifugation when shearing red blood cells (Schrier et al., 1971). MVs are ∼100–1000 nm in size, although this range can vary and overlap with ApoBDs (Menck et al., 2020). MVs are also known as shedding vesicles, shedding MVs, or ectosomes in the literature because they bud outwards and fuse with the plasma membrane of cells (Anand et al., 2019). While there is some debate on the mechanisms of cellular MV secretion, a canonical method involving budding and ectocytosis of the vesicle upon fusion with the parent cells’ plasma membrane has been described (Stahl and Raposo, 2019). This process is proposed to be facilitated by intracellular EV shuttling machinery that is evolutionarily conserved across species, such as SNARE (Kloepper et al., 2007), or endosomal sorting complex required for transport (ESCRT) (Leung et al., 2008). MVs are typically characterized by expression of Annexin A1, Integrins, and CD62. In addition to exosomes, MVs have also been proposed to drive intercellular communication through the cargo they carry, including proteins, lipids, and nucleic acids (Lv et al., 2019). MVs are released by both healthy and malignant tissues, and are present in a variety of biological matrices, such as urine, saliva, and plasma (Ratajczak et al., 2006; Yeri et al., 2017; Bruno et al., 2019). MV release can be impacted by extracellular stimuli, for example, a hypoxic tumor environment leads to increased MV release (Wang et al., 2014). MVs also carry ADP-ribosylation factor 6 (ARF6) and ESCRT family that help facilitate cell-cell communication in tumor cells (Muralidharan-Chari et al., 2009).

#### Apoptotic bodies (ApoBDs)

ApoBDs are formed by cells undergoing apoptotic cell disassembly and can range from 500 nm to 2 µm in size. They have been described as “little sealed sacs” that are a hallmark of apoptosis and thought to function as “garbage bags” until recent discoveries revealed their potential utility as delivery tools (Battistelli and Falcieri, 2020). ApoBDs contain diverse cellular components and have been shown to facilitate the transfer of DNA and protein between cells and facilitate viral propagation and mount an immune response (Holmgren et al., 1999; Li X. et al., 2021). Because ApoBDs are formed *via* cellular blebbing after programmed cell death, the surface protein landscape of an ApoBD can vary but will typically retain markers from their cell of origin. Formation of ApoBDs involves protein kinases including Rho-associated kinase (ROCK1) (Sebbagh et al., 2001), myosin light chain kinase (MLCK) (Mills et al., 1998), and the plasma membrane channel pannexin 1 (PANX1) (Poon et al., 2014). Due to their apoptotic origin, phosphatidylserine has been identified as a common surface marker for ApoBDs (Atkin-Smith et al., 2015). After ApoBDs are released, they can be targeted to be phagocytosed by neighboring cells such as macrophages and degraded by phagolysosomes to prevent secondary necrosis (Erwig and Henson, 2008). This led to a study investigating an apoptotic body-based vehicle harboring R848 apoptotic body-based nanoparticles containing IR820-conjugated antibodies, effectively using ApoBDs as a delivery vehicle to breast tumors in a mouse model (Sheng et al., 2022). In addition to this, ApoBDs can be taken up by other phagocytes such as dendritic cells, which can display apoptotic antigens on their surface to facilitate an immune response (Tixeira et al., 2020). Thus, ApoBDs can potentially cross-activate the innate and adaptive immune system. Although not much is known about the biological role of ApoBDs, they have been investigated in the use of vaccine development and immunotherapy (Phan et al., 2020).

### EV separation and enrichment

With an increase in publications investigating the function of EVs in the last decade, the International Society for Extracellular Vesicles (ISEV) has proposed Minimal Information for Studies of Extracellular Vesicles (MISEV), a comprehensive list to guide EV-related research (Théry et al., 2018). The guidelines include recommendations for EV isolation, characterization, and how to perform EV-associated functional assays. Over 380 ISEV members who are emerging experts in the EV field contributed to the MISEV 2018 guidelines, which currently has over 5000 citations. The group convenes at least annually to update the MISEV as necessary and provide EV researchers the most up-to-date information regarding methods for EV isolation and characterization, as well as EV-associated functional assays and other advances in EV-focused technology. This has helped minimize discrepancies in the field. A current updated version is being developed, as new information has accumulated since the release of the 2018 guidelines. Technical limitations in isolating EVs from cells and blood have hindered progress and limited the potential of using EVs for therapeutic and diagnostic purposes. However, understanding the role of EVs in cancer onset and progression has grown in large part because of more comprehensive EV characterization developed over the past decade.

As described above, technical limitations have impaired the isolation and characterization of EVs, particularly because of overlapping characteristics between EV subpopulations. This has limited the clinical applications and interpretation of data, as the integrity and purity of EVs varies depending on the isolation method. Table 1 compiles a selection of methods currently used, including their specific uses, as well as the advantages and disadvantages of each method. An important consideration in EV isolation is that co-isolated materials, such as protein aggregates, lipoproteins, and viruses may be associated with observed EV functions (Holcar et al., 2021). This highlights the importance of choosing multiple, diverse techniques to characterize EVs, including negative markers to track co-isolated non-EV components. The EV-TRACK consortium assembled a crowdsourcing knowledge base to standardize EV practices and methodologies. Surprisingly, 17% of experiments submitted did not include characterization of EVs, with 39% of experiments being limited to particle analysis, such as NanoSight analysis (Van Deun et al., 2017). The number of particles can be determined by using nanoparticle tracking analysis (NTA) or by using flow cytometry for large EVs (Atkin-Smith et al., 2015; Cointe et al., 2017). The MISEV2018 outlines suggested steps for selecting protein markers to characterize EVs. Technical limitations to accurately track EV secretion in real time have made it difficult to determine the levels of EVs released from tumor cells. To address this need, Kilic et al. developed a label-free electrochemical sensor to determine secretion of EVs from human MCF7 breast epithelial cells under hypoxic conditions (Kilic et al., 2018). This method relies on the use of the transmembrane CD81 biomarker and was shown to provide more reproducible results compared to ELISA. Additional methods commonly employed to characterize EVs have been summarized in Table 2.

**TABLE 1:** Methods for enriching and isolating EVs. Various techniques have been developed for the isolation and enrichment of EVs. Each approach has its advantages and disadvantages that can aid in deciding the appropriate approach.

Method	Approach	Advantages	Disadvantages	References
Density-gradient ultracentrifugation (dUC)	Combines centrifugal force and density gradient mediums including iodixanol and membranes to separate EVs based on buoyant density. Centrifugation is typically 100,000–120,000 *g* for 16 hrs	Can be used for large volumes of conditioned media	Co-isolation of non-EV particles and loss of EVs can occur	Zhang et al. (2014), Brennan et al. (2020)
Immunoaffinity-based capture	Uses antibodies conjugated to particles to selectively bind EV surface markers to purify desired EVs	Highly specific to select for cancer-derived EVs, compatible with light scattering flow cytometry	Not standardized and requires EV-specific surface markers	Brett et al. (2017), Morales-Kastresana et al. (2019)
Polymer based precipitation	Uses polymer-based particles (e.g. polyethylene glycol, PEG) to pellet EVs out of solution	Time efficient with various commercially available kits	Aggregation and coprecipitation with non-exosomal materials	Brown and Yin (2017)
Size exclusion chromatography (SEC)	Uses elution time from bead column to distinguish and separate EVs based on size	High integrity of isolated EVs, and low-cost columns are available	Not specific, and only uses size to separate, susceptible to contamination by non-EV particles	Palmieri et al. (2014), Gámez-Valero et al. (2016)
Tangential-flow filtration (TFF) for EV isolation	Uses a tangential flow filter to pass EV-containing liquid through a membrane pore to capture EVs	Allows supernatant to be both concentrated and filtered simultaneously and has been used in 3D culture	Requires secondary filtration to increase EV yield	Busatto et al. (2018), Haraszti et al. (2018), Paterna et al. (2022)
Ultra-centrifugation	Uses high speed (100,000-110,000 *g* for 16–18 hrs) to pellet EVs from supernatant	Has been reported to have low co-isolation of proteins	Labor intensive and low throughput; recovery of EVs is highly variable, and may damage EVs	Gardiner et al. (2016), Momen-Heravi (2017), Takov et al. (2019)

**TABLE 2:** Methods of EV characterization. It is important to characterize isolated EVs to determine the purity and integrity of EVs. Various techniques can be used depending on downstream analysis.

Method	Description	References
Transmission electron microscopy (TEM)	Can measure single EVs using nanometer resolution, and is often used to determine if a sample is pure	Rikkert et al. (2019)
Nanoparticle tracking analysis (NTA)	Widely used to determine size and particle distribution of EVs by shining a laser beam through an EV suspension mixture. The NanoSight Ltd manufactures machines that are used for this method	Dragovic et al. (2011), Maas et al. (2015), Gardiner et al. (2016)
Dynamic light scattering (DLS)	Also known as photon correlation spectroscopy, uses photon detectors to produce a distribution plot of particles	Palmieri et al. (2014)
Flow cytometry	Used for characterization and detection of surface proteins on EVs	Chiang and Chen (2019)
Enzyme-linked immunosorbent assay (ELISA)	Used for detection and quantification of EV proteins. Common capture antibodies include CD63 and CS81 followed by a secondary labeled antibody	Hartjes et al. (2019)
Western blot	Used to determine presence of proteins on EVs. According to the MISEV2018 at least one transmembrane protein (CD9, CD63, CD81) and one cytosolic protein (TSG101, Alix, syntenin) should be used	Théry et al. (2018)

In the past decade, a wide array of methods for EV isolation have been developed and made commercially available. However, many of these methods lack standardized techniques which can impact EV characterization, making it difficult to determine to precise role of EVs in cancer onset and progression. Therefore, each method must be carefully evaluated to determine its impact on downstream assays. For the isolation of exosomes from plasma and serum, differential ultracentrifugation (dUC) and size exclusion chromatography (SEC) are the two predominant methods. dUC utilizes centrifugal force to separate EVs and has become a widely used method for EV isolation (Théry et al., 2006; Gardiner et al., 2016). Typically, low speed centrifugation (<10,000 *g*) is used to remove cells and cell debris (1,000 *g*), while high speeds (100,000–200,000 *g*) are used for final EV separation (Musante et al., 2013; Wang F. et al., 2021). This method has been used to concentrate EVs without the use of harsh chemicals (Théry et al., 2006). Additional washing steps can increase EV purity, but may reduce the final EV concentration. SEC, sometimes referred to as gel filtration, separates EVs by size based on their ability to penetrate gel pores during a stationary phase; it has been used to isolate exosomes from various biological fluids (Sidhom et al., 2020). Currently, there are various exosome isolation kits that employ this technique, including SmartSEC™ Single for EV Isolation (System Biosciences), qEV (Izon Science), PURE-EVs (Hansa Biomed), Qev (Izon Science), and exoEasy kit (Qiagen). Izon Science manufactures 35 nm and 70 nm qEV columns that have been widely used due to their efficiency, ease of use, and availability. SEC columns can be used to isolate EVs from plasma, serum, and culture media. Since this method commonly uses a phosphate buffer at pH 7.4 during the separation phase, there are no harsh chemical reagents needed, making this an attractive method for therapeutic and diagnostic applications (Sidhom et al., 2020). One downside is the potential for small non-exosomal vesicles, large protein aggregates, and lipoproteins that may contaminate the EV population (Brennan et al., 2020). Additionally, the cost of the commercially available columns can be upwards of $500.

Polymer precipitation is an inexpensive and simple method for isolating EVs that commonly uses polyethylene glycol (PEG) as a precipitating agent. Various commercially available kits using this approach have been made, including ExoQuick (System Biosciences), ExoPrep (HansaBioMed), and Exosome Isolation kit (Exiqon). Protocols for PEG-based approaches to EV isolation have also been described previously (Brown and Yin, 2017). Although this method is quick and results in a high yield, a major limitation is the co-precipitation of proteins and non-EV associated nucleic acids. Co-precipitation of albumin, apolipoprotein E, and immunoglobulins make mass spectrometry, proteomic analysis, and RNA analyses difficult (Van Deun et al., 2014; Lobb et al., 2015). Ultracentrifugation has commonly been used to separate proteins and other contaminating components by size. This method takes advantage of a membrane with different sized pores to capture EVs, while other molecules pass through the filter (Xu et al., 2015; Xu et al., 2017). A disadvantage to this method is reduced sample recovery when the filter becomes clogged, and co-isolation of products of similar size to your target. This method can also be time-consuming depending on starting material volume (Merchant et al., 2010; Cvjetkovic et al., 2014; Taylor and Shah, 2015). Charge-based precipitation uses the negative charge of EVs under physiological conditions to precipitate EVs in the presence of a positively charged protamine (Deregibus et al., 2016).

Immunoaffinity-based capture methods use antibodies to bind to EV surface proteins to enrich the EV population. Many immunoaffinity-based methods are still being developed and have not been standardized; however, they have yielded promising results and recent studies suggest that immunoaffinity-based methods may be superior for EV purification compared to commercially available EV isolation kits (Brett et al., 2017). Although this technology has been successful in the isolation of EVs from melanoma (Sharma et al., 2018), colon cancer (Tauro et al., 2012), and pancreatic cancer (Wang J. M. et al., 2021), the limitations of relying on specific protein targets make it difficult to implement for a variety of EVs from cancer cells. Because immunoaffinity-based capture methods rely on antibodies to bind to EVs, cancer specific protein markers must be found for targeting cancer-derived EVs, therefore this remains a significant challenge with this approach (Ruhen and Meehan, 2019). However, there are studies underway aiming to characterize surface markers present on EVs specifically isolated from cancer patients (Nazimek and Bryniarski, 2020; Hu et al., 2022). For example, immunoaffinity-based methods have been used to isolate melanoma-derived exosomes using a monoclonal antibody targeting the CSPG4 epitope expressed on EVs derived from melanoma cells (Sharma et al., 2018).

These techniques also have their weaknesses which can influence EV yield and integrity. For example, SEC separates based on size, which can lead to non-specific, co-isolation of similar-sized molecules. Additionally, viscosity in the EV solution can alter EV yield when using ultracentrifugation (Brown and Yin, 2017). Finally, because immunoaffinity-based capture relies on antibodies or magnetic beads, the process of eluting EVs from antibodies conjugated to magnetic beads may lead to processing issues, such as maintaining the integrity of the EVs during the elution process (Buschmann et al., 2021).

### EV cargo

Cancer-derived EV cargo varies depending on cancer type and stage. This cargo may include a diverse range of nucleic acids, proteins, and lipids that can be a source of pro-tumorigenic and anti-tumorigenic signaling molecules. Here, we will summarize differences in protein and RNA EV cargo analyzed in samples collected from breast, prostate, and glioma patients and models.

#### RNA

Key findings between 2006 and 2008 highlighted the role of RNAs in EVs and launched studies looking at RNA as EV cargo (Baj-Krzyworzeka et al., 2006). MicroRNAs (miRNAs or miRs) are approximately 20–25 nucleotides long and regulate gene expression through post-transcriptional modifications. In mammals, most protein-coding RNA sequences contain at least one miRNA-binding site with 57% of genes containing conserved miRNA targets (Friedman et al., 2009). Therefore, the miRNA content packaged in cancer-derived EVs plays a critical role in regulating RNA translation in the TME. Currently, there are more than 2,600 miRNAs that have been identified in humans (Kozomara and Griffiths-Jones, 2014).

In 2007, Valadi et al. found that miRNAs are present in biological fluids as circulating free miRNAs and in lipid bound structures, including EVs (Valadi et al., 2007). This study opened avenues for research focused on EV-associated miRNAs and their function in cancer pathogenesis. Cancer-derived EVs have associated miRNAs that play a role in intercellular communication (Dominiak et al., 2020). Additional RNA molecules including transfer RNAs (tRNAs), long non-coding RNAs (lncRNAs), and viral RNAs have also been found in EVs (Valadi et al., 2007). The packaging of RNA molecules in EVs is regulated in part by RNA binding proteins (RBPs), which are proteins that recognize specific miRNA motifs. RBPs can exclude miRNAs from EV packaging by binding to specific sequences in the miRNAs. This is exemplified by increased RNA packaging into EVs by silencing of the RBP hnRNPH1 (Statello et al., 2018). A selection of RNAs and their association with individual cancers can be found in Table 3. RNA contained within EVs can have diverse effects on cancer including interacting with the TME and driving tumor growth, which is reviewed below.

**TABLE 3:** Selection of cargo in cancer-derived EVs. EVs arise from a variety of cancers that contain specific RNA and proteins molecules as described.

Nature of cargo	Name	Cancer	References
RNA	miR-335	Hepatocellular	Wang et al. (2018)
	miR-206	Osteosarcoma	Monfared et al. (2019)
	miR-193a	Colon	Zhang et al. (2020)
	miR-144–3p	Cervical	Ying et al. (2020)
	miR-125b	Hepatocellular	Meng et al. (2021)
	miR-21	Colorectal	Hong et al. (2009)
	miR-182	Prostate	Mihelich et al. (2016)
	miR-183	Prostate	Mihelich et al. (2016)
	miR-222	Breast	Ding et al. (2018)
	miR-155	Melanoma	Dror et al. (2016)
	miR-211–5p	Melanoma	Lunavat et al. (2017)
	hTERT mRNA	Lung and pancreatic	Gutkin et al. (2016)
	miR-100–5p	Prostate	Sánchez et al. (2016)
	miR-21–5p	Urothelial	Matsuzaki et al. (2017)
	miR-409	Prostate	Josson et al. (2015)
	TMPRSS2:ERG	Prostate	Motamedinia et al. (2016)
	miR-134	TNBC	O’Brien et al. (2015)
Long non-coding RNA	SNHG3	Colorectal	Zhao et al. (2022)
	LOC441178	Esophageal	Jiangmu Chen et al. (2022)
Protein	FAK	Breast	Vinik et al. (2020)
	MEK1	Breast	Vinik et al. (2020)
	Fibronectin	Breast	Vinik et al. (2020)
	EGFR	Breast	Galindo-Hernandez et al. (2013)
	ADAM10	Breast	Galindo-Hernandez et al. (2013)
	Survivin	Breast	Khan et al. (2014)

#### Protein expression in cancer-derived EVs

In addition to RNA, proteins are also a component of EV cargo (Table 3). EVs can serve as protein transporters and proteins contained within EVs can correspond with the molecular cancer subtype. For example, the epidermal growth factor receptor (EGFR) is amongst the most important signaling pathways involved in regulating growth, proliferation, and differentiation in mammalian cells (Wee and Wang, 2017). Mutant EGFR, EGFRvIII has a truncated extracellular domain and is associated with increased tumorigenicity mediated by EGFRvIII kinase activity and tyrosine autophosphorylation at the C-terminus. This mutant is expressed in nearly 60% of GBM cases (Cavenee, 2002). The addition of EVs containing this mutant receptor transferred the oncogenic phenotype from mutant U373 glioma cells to indolent cells. This presented initial evidence of EV-mediated horizontal transference of oncogenic phenotypes. This was later demonstrated in the human breast cancer cell line MDA-MB-231 and human glioma cell line U87. Al-Nedawi et al. first revealed horizontal transference of the mutant receptor, EGFRvIII, from mutant U373 human glioma cells to wildtype cells (Al-Nedawi et al., 2008). They found oncogenic EGFRvIII expression in indolent glioma cells following treatment with EVs derived from U373 glioma cells secreting the mutant EGFRvIII receptor. Metastasis-related proteins are expressed in EVs from epithelial MCF-7, the highly aggressive triple negative breast cancer (TNBC)-derived MDA-MB-231, and epithelial T47D cell lines including the interleukins (IL-) IL-6, IL-8, IL-12, vascular endothelial growth factor (VEGF), FGF basic, G-CSF and GM-CSF (Dalla et al., 2020). The MDA-MB-231 and T47D derived EVs contain moderate amounts of tenascin, a protein that induces epithelial-mesenchymal transition (EMT) (Dalla et al., 2020). EVs derived from MDA-MB-231 cells have surface expression of CSF-1, (colony stimulating factor-1) (Tkach et al., 2022). Additionally, TβRII is found in TNBC cells with increasing metastatic potential (Xie et al., 2022). The authors found that EVs from MDA-MB-231 and 4T1 TNBC lines contain TβRII, and when delivered to CD8^+^ T cells, leads to the activation of SMAD3 and subsequent CD8^+^ T cell exhaustion. In prostate cancer (PCa), androgen receptor (AR) and truncated AR (AR-7) are found in LNCaP and PC3 PCa human cell lines and can be transferred to AR-null cells (Read et al., 2017). CUB domain-containing protein is expressed in PCa-derived EVs (Sandvig and Llorente, 2012; Mizutani et al., 2014; Read et al., 2017). Exosomes isolated from the plasma of melanoma patients had increased levels of CD63 and Caveolin-1 (tumor-associated marker) compared to healthy donors (Logozzi et al., 2009). Myosin-9 is present in EVs derived from MDA-MB-231 cells with decreased signal-induced proliferation-associated 1 (SIPA1) expression (Feng et al., 2022).

### EVs and the TME

The TME represents the diverse ecosystem surrounding a tumor that contains numerous cellular and non-cellular components that play a large role in cancer onset and progression and is comprised of a diverse number of proteins (elastin, collagen, laminin, and fibronectin), growth factors (transforming growth factor B and VEGF), sugars, and enzymes, all of which form a dynamic structural network (Kular et al., 2014; Karamanos et al., 2021). Cancers remodel their environment to support their growth through modification of the ECM *via* cross-linking and immunosuppression, degradation of the ECM, and deposition of ECM components (Winkler et al., 2020). A canonical characteristic of solid tumor development is altered ECM density and composition, which increases tissue stiffness and may influence clinical outcomes (Seewaldt, 2014). As such, targeting the stiff ECM has been proposed to improve the efficacy of cancer therapies (Jiang et al., 2022).

EVs can play a critical role in the TME by mediating signaling between cancer cells and TME cells (fibroblasts and macrophages) and priming the TME to support metastasis. For example, cancer cell-derived EVs can confer oncogenic properties to surrounding non-cancerous cells by altering their phenotypes. Additionally. monocytes treated with MVs derived from pancreatic, lung, and colorectal cancer led to their pro-inflammatory polarization, characterized by enhanced anti-tumor activity *in vitro* (Baj-Krzyworzeka et al., 2007)*.* However, while present, the exact biological function and role of ApoBDs in the TME has not been fully characterized, representing a gap in knowledge that requires further investigation.

As mentioned in the EV separation and enrichment section, it can be difficult to distinguish and isolate subpopulations of EVs. For the subsequent sections, the nomenclature of EVs will follow what the authors of the presented studies have used.

#### Stroma-to-tumor communication as a driver of cancer progression

The stroma is primarily composed of fibroblasts, immune cells, and the extracellular matrix (ECM) and provides a structural and connective role. The stroma within the TME promotes metastasis and cancer progression. However, the mechanisms and functional role of the stroma have only recently been explored. Fibroblast-derived exosomes promote breast cancer cell protrusive activity and mobility *via* Wnt-PCP signaling, providing evidence of pro-tumorigenic stroma-to-tumor communication (Luga et al., 2012). Stromal triggering of the NOTCH-MYC pathway by breast cancer cells resulted in stromal EVs containing increased unshielded RNA component of signal Recognition Particle 7SL1 (RN7SL1) (Nabet et al., 2017). RN7SL1 is typically shielded by RBP SRP9/14, but loss of these RBPs leads to inflammation and activation of its pattern recognition receptor, retinoic acid-inducible gene I (PRR RIG-I), leading to therapeutic resistance, tumor growth, and metastasis.

##### EVs mediate intercellular communication in the TME

Integrin-β3 (ITGβ3), a surface integrin, facilitates the endocytosis of EVs into MDA-MB-231 breast cancer cells and its ablation decreased EV uptake (Fuentes et al., 2020). Loss of CD9 expression also impaired EV uptake into pancreatic ductal adenocarcinoma cells, causing decreased migration and EMT (Nigri et al., 2022). Colorectal cancer-derived MVs also increased angiogenesis in the TME by increasing endothelial cell proliferation (Hong et al., 2009). PCa derived miRNAs miR-100, miR-21, and miR-139, increases expression of receptor activator of nuclear factor kappa-B ligand (RANKL) and metalloproteinases in cancer-associated fibroblasts, increasing their proliferation, differentiation, and migration (Sánchez et al., 2016). EVs derived from the highly aggressive human TNBC cell line MDA-MB-231 and U87 human glioma cells can impart the transformed characteristics of tumor cells onto normal fibroblasts and epithelial cells (Antonyak et al., 2011).

#### EVs restructure the TME to establish a pre-metastatic niche

EVs can restructure the TME to establish a pre-metastatic niche (PMN) which is an environment that is favorable and conducive to enhanced tumor growth and distant metastasis (Peinado et al., 2017). Common factors that work to establish the PMN include VEGF, pro-inflammatory cytokines such as tumor necrosis factor alpha (TNFα), IL-6, transforming growth factor β (TGF-β), interferon gamma (IFN-γ), and IL-1β (Li et al., 2020). EV uptake promotes angiogenesis *via* induction of endothelial dysfunction by upregulating the expression of angiogenesis genes and IL-8, a pro-inflammatory cytokine that promotes cancer metastasis (Nazarenko et al., 2010; Singh et al., 2013; Todorović-Raković and Milovanović, 2013). When EVs derived from highly metastatic 4THM (4T1 heart metastases) murine breast cancer are infused into mice bearing less metastatic EMT6 breast cancer tumors, the EMT6 tumors also become highly metastatic, a process thought to be facilitated through the enhanced secretion of IL-6 (Gorczynski et al., 2016). Additionally, some cancer-derived EVs express programmed death ligand 1 (PD-L1) on their surface, an inhibitory molecule that binds to its receptor (programmed-cell death protein 1 (PD-1)) and suppresses CD8^+^ cytotoxic T cell function (Chen et al., 2018). This causes immunosuppression and transforms the TME into a PMN that promotes tumor growth, an effect observed in breast cancer and leukemia (Yang et al., 2018; Li C. et al., 2021; Gargiulo et al., 2022). EVs from breast tumors polarize macrophages to a M2 anti-inflammatory, pro-tumor phenotype *via* metabolic remodeling of macrophages, as well as upregulation of glycolysis and PD-L1 expression. Notably, these breast cancer derived EVs migrated to the lung, where they recruited myeloid-derived suppressor cells and PD-1 expressing T cells to prime the lung for metastasis *via* immunosuppression (Morrissey et al., 2021). Thus, EVs are implicated in hampering both the innate and adaptive arms of immunity, creating a highly favorable environment for cancer metastasis.

Cancers can promote their growth through paracrine signaling to remodel the TME to support tumorigenesis. In liver cancer, EVs derived from hepatic stellate cells were preferentially taken up by hepatocellular carcinoma cells, contributing to their growth and progression by upregulating glycolysis, a phenotype recapitulated *in vitro* and *in vivo* in mouse models (Chen Q. T. et al., 2022). EVs also interact with leukocytes (particularly macrophages) in the TME to promote metastasis (Yang et al., 2022). These macrophages, known as metastasis-associated macrophages, upregulate CD36 expression, which can drive the uptake of lipid-rich EVs. This uptake polarizes macrophages towards a M2, pro-tumor, anti-inflammatory phenotype to reshape the TME into one conducive to further growth and progression.

#### Cancers use EVs to prime distal sites for metastasis

Some cancers use endocrine signaling *via* EVs to distally promote their growth by priming new sites for invasion and metastasis. Mechanisms by which this occurs include triggering the release of inflammatory factors, hampering immunosurveillance, promoting angiogenesis, and increasing vascular permeability (Chen X. et al., 2022). Cancer-derived EVs trigger the release of IL-6, a pro-inflammatory cytokine, by bone-marrow derived macrophages by activating the IL-6-STAT3 signaling cascade (Ham et al., 2018). Primary colorectal tumors release integrin beta-like 1 (ITGβL1)-rich EVs into circulation to activate fibroblasts in distal organs to induce a pro-inflammatory environment filled with activated fibroblasts, the combination of which helps cancers grow and spread (Ji et al., 2020). This corroborated an earlier study showing that specific combinations of integrins were more highly associated with metastasis to certain organs, whereas EVs rich in other integrin combinations were prone to spread to other organs (Hoshino et al., 2015). This study included both exosomes isolated from mouse and human lung, liver, and brain-tropic tumor cells. Clinical relevance of this study shows that exosomes harboring integrins may be used to predict organ-specific metastasis. Furthermore, secreted nucleoside diphosphate kinase A and B (NDPK) and phosphotransferase expression and activity have been shown to be elevated in breast cancer-derived EVs and increase endothelial cell migration and cause vascular leakage, both pro-tumorigenic effects (Duan et al., 2021). EVs containing miR-181c and miR-105 destroy vascular endothelial barriers, such as the BBB (Hatzidaki et al., 2019a; Hatzidaki et al., 2019b; Guo X. et al., 2020) and provide an alternative avenue of metastasis (Zhou et al., 2014). Taken together, this provides a potential tool to aid prediction of where certain tumors may be more likely to metastasize to prior to overt spread by characterizing the cargo within EVs and identifying their source.

#### EVs hamper cancer growth and progression through immune system regulation

EVs derived from MDA-MB-231 and BT-549 TNBC cells express CSF-1, which polarizes macrophages towards a pro-inflammatory, anti-tumor state, reinforcing a competent immune microenvironment that is associated with improved prognosis in patients with TNBC (Tkach et al., 2022). EVs derived from dendritic cells express major histocompatibility complex (MHC) I and II, as well as T-cell costimulatory molecules, the combination of which are sufficient to activate cytotoxic T-cells *in vivo* and inhibit the growth of mouse mastocytoma and mammary carcinoma (Zitvogel et al., 1998). This phenotype was recapitulated with both CD4^+^ helper T and CD8^+^ cytotoxic T cells in mouse models of brain cancer when dendritic cell EVs were loaded with chaperone-rich cell lysates, a robust source of immune activation (Bu et al., 2015). Similar findings were reported by Segura et al., which reported that exosomes from mature dendritic cells are critical for inducing potent antigen-specific T cell response and activation *in vitro,* whereas those from immature dendritic cells are unable to trigger as robust a response (Segura et al., 2005; Lugano et al., 2020). Proteomic analysis of EVs derived from mature dendritic cells found enrichment of MHC II, B7.2, ICAM-1, and MFG-E8, which serves to prime naïve T cells for activation (Segura et al., 2005). Although these studies focused on adaptive immunity, these findings have been recapitulated in the innate immune system as well. EVs derived from mature dendritic cells helped to trigger NK cell activation and proliferation *in vivo via* NKG2D and IL-15RA, respectively (Viaud et al., 2009).

### Targeting EV synthesis and release

With the advancement of gene manipulation and our understanding of the plethora of signaling pathways and complexes required for EV biogenesis, transport, and secretion, there have been promising studies leading the effort to identify methods of targeted depletion or inhibition of EVs to mitigate their impact. For example, RNA interference (RNAi)-based silencing of the machinery required for ESCRT function, such as STAM1, TSG101, or HRS, led to a dramatic reduction in EV secretion, whereas silencing of Alix increased EV secretion (Colombo et al., 2013). EV-associated PD-L1 was also significantly lowered upon RNAi-mediated disruption of HRS (Chen et al., 2018). In addition to the ESCRT pathway, the Syndecan-Syntenin-Alix pathway is also involved in EV synthesis. Disruption of this pathway using RNAi led to a significant decrease in EV release (Baietti et al., 2012). ADP ribosylation factor 6 (ARF6) and phospholipase D2 (PLD2) are critical regulators of EVs expressing Syntenin-Alix, the loss of either of which significantly reduced EV formation and secretion (Ghossoub et al., 2014). In addition to EV synthesis, there are several pathways that regulate EV trafficking, prior to secretion. Rab GTPases are a class of proteins that are heavily involved in membrane trafficking, EV formation, and EV transport (Stenmark, 2009). When components of this family such as Rab27a or Rab27b were knocked down *via* RNAi, a significant ablation of EV secretion was observed in head and neck squamous cell carcinoma (Hoshino et al., 2015) and cervical cancer (Ostrowski et al., 2010). The loss of these components also suppressed EV transport within the cell and to the cell membrane, preventing fusion and subsequent release (Ostrowski et al., 2010). In addition to RNAi, knockout of Rab27a *via* CRISPR-Cas9 inhibited EVs secretion (Poggio et al., 2019). The SNARE protein family is largely responsible for mediating EV fusion with the membrane and eventual exocytosis (Salaün et al., 2004). Downregulation of syntaxin-6, a member of the SNARE family, *via* RNAi decreased EV secretion in PCa cells (Peak et al., 2020).

Beyond gene manipulation, there have been pharmacological advances made in targeting EVs both *in vitro* and *in vivo.* Notably, GW4869, a non-competitive inhibitor of sphingomyelinase (SMase), a protein involved in EV budding, is a top candidate for targeting EV biogenesis and secretion (Essandoh et al., 2015). There have been a plethora of *in vitro* and *in vivo* studies involving GW4869 that have validated it as an effective EV depletion approach. For example, GW4869 ablates EV secretion in breast (Yang et al., 2018), skin (Montermini et al., 2015; Matsumoto et al., 2017), and bladder cancers (Ostenfeld et al., 2014). *In vivo* mice bearing B16BL6 melanoma tumors treated intratumorally with GW4869 showed significant ablation of tumor growth and decreased EV secretion (Matsumoto et al., 2017). To date, a handful of GW4869 delivery methods have been studied and validated, including a hyaluronic acid-based nanoplatform (Wang G. et al., 2021). However, a significant caveat is that because GW4869 non-competitively targets SMase, it is unable to selectively prevent the synthesis and release of cancer-derived EVs, therefore this compound also depletes non-cancer EVs. Additionally, SMase has been characterized to play a significant role in other biological processes throughout the body, requiring more specialized targeting to localize GW4869 to cancer-derived EVs.

### Exploiting EVs to target the TME

Given the plasticity of the TME, it is advantageous to exploit EVs as a therapeutic approach to fight cancer. This can occur in a few different ways including using EVs as a drug delivery system to directly target tumors or as a method of remodeling the TME. This therapeutic avenue is largely attractive due to the biological nature of EVs, conferring stable bioavailability as well as distribution *in vivo,* and EVs can carry a diverse range of different cargo (Kang et al., 2021). In addition to this, solid cancers can exhibit a phenomenon known as the enhanced permeability and retention effect (EPR), which describes increased accumulation of nanoparticles in tumors compared to regular tissues. As EVs are a type of nanoparticle, it is plausible that they may be subject to EPR (Phillips et al., 2021). EPR is observed in various solid tumors, and is characterized by abnormal tumor vasculature, tumor permeability, and a lack of effective lymphatic drainage (Wu, 2021). The leaky tumor vasculature allows for nanoparticles to extravasate through surrounding blood vessels (Maeda et al., 2013). Together, these function to help nanoparticles persist and accumulate in tumors, allowing for retention and direct delivery to the site of interest (Wu, 2021). These characteristics make EVs an attractive therapeutic avenue that can be adapted to different treatment approaches.

### EVs in drug delivery

EVs have potential use as drug carriers for targeted delivery to tumors (Herrmann et al., 2021). Cancer-derived EVs have the unique ability to identify sites of early neoplasia, which could potentially allow for early detection of sites that may be prone to developing cancer and as a tool for delivering treatments prior to overt disease onset (Garofalo et al., 2021). EVs have been identified as carriers of chemotherapeutics including paclitaxel and doxorubicin which target murine breast cancer lung metastases (Haney et al., 2020). Paclitaxel is also incorporated into EVs from stromal cells which then get secreted into the extracellular space, producing an additional source of drug-loaded EVs to synergize and maximize treatment efficacy (Pascucci et al., 2014). This has also been demonstrated with an ERK inhibitor; EVs loaded with this molecule are taken up by TNBC cells, significantly decreasing migration and proliferation but not overall viability (Hatzidaki et al., 2019a; Hatzidaki et al., 2019b; Guo Y. J. et al., 2020). EVs secreted by brain cell lines can be engineered to carry potent anti-cancer drugs and can successfully cross the BBB, a prominent hindrance to successful treatment delivery to the brain (Yang et al., 2015). In a zebrafish model, EVs loaded with doxorubicin or paclitaxel can localize and enter the brain, revealing a novel source of drug vehicles that can help overcome challenges associated with the BBB (Zhao et al., 2021). Murine colorectal cancer studies of doxorubicin-loaded exosome-mimetic nanovesicles identified their ability to traffic to the tumor and significantly reduce tumor growth with minimal unwanted side effects (Jang et al., 2013). Similar findings have been reported in doxorubicin-loaded tumor-derived EVs for their ability to significantly inhibit the growth of colorectal tumors *in vivo* (Nguyen et al., 2022)*.* Furthermore, ApoBDs derived from apoptotic cancer cells deliver residual chemotherapeutic drugs to neighboring cells (Zhao et al., 2021).

In addition to chemotherapy drugs, EVs have also been engineered to contain anti-tumor RNAs. EVs containing anti-tumor miRNAs, such as miR-21 and miR-451a, induce apoptosis in an *in vitro* model of liver cancer (Pomatto et al., 2019). As reviewed above, there have been many studies involving EVs containing miRNAs and their potent anti-tumor effects; therefore, this creates an exciting treatment modality allowing for the direct delivery of anti-tumor miRNAs as a possible therapeutic avenue.

### Using EVs to modulate the immune system

An *in vivo* study of melanoma in mice successfully generated EVs containing antigenic peptides enveloped by an erythrocyte membrane, to facilitate uptake into immune cells (Guo et al., 2015). Preliminary *in vitro* experiments demonstrated that these EVs retain their antigenic content and the erythrocytic nature of the membrane promoted their uptake. The administration of these EVs to mice with melanoma triggered a significant release of IFN-γ and upregulation of activation of cytotoxic CD8^+^ T cells (Guo et al., 2015; Smith et al., 2017). Tumor-derived EVs can be taken up by monocytes, transforming them to immuno-suppressed cells and permitting the cancer cells to evade immune response (Luong et al., 2021). EVs evade immune response when monocytes take up these EVs and increase the expression of the anti-inflammatory cytokines and mediators: IL-10, TGF-β, arginase, and iNOS (Luong et al., 2021). Tumor derived EVs that contain the long non-coding RNA LOC441178 profoundly suppressed esophageal carcinoma progression, primarily by preventing M2 macrophage polarization (Chen J. et al., 2022). Additionally, the uptake of these EVs also has a negative effect on the expression of IL-12 and TNF-α (Plebanek et al., 2018). Other immune cell types that have reported success with EV-based modification include myeloid-derived suppressor cells (MDSCs) *via* high-density lipoprotein laden EVs binding scavenger-receptor type B-1 (SCARB1) on MDSCs (Plebanek et al., 2018), macrophages *via* selective targeting of M2 macrophages and tumor-associated macrophages (Wang et al., 2019; Chen et al., 2020; Chen et al., 2021), *via* induction of M1 polarization (Wang et al., 2022), or *via* genetic reprogramming of macrophages using nanocarriers packed mRNA to induce transcriptional level changes (Zhang et al., 2019). Treatment of monocytes with MVs derived from pancreatic cancer, lung cancer, and colorectal cancer led to a pro-inflammatory polarization, characterized by enhanced anti-tumor activity *in vitro* (Baj-Krzyworzeka et al., 2007)*.* Conversely, Wieckowski et al. found that MVs derived from cancer cell lines suppressed a T cell-mediated immune response by increasing primary regulatory T cell expansion and apoptosis of primary CD8^+^ T cells (Wieckowski et al., 2009). Wang et al. subsequently demonstrated that administration of nanoparticles containing inhibitory compounds was found to target both the innate and adaptive immune system by reducing the expression of PD-L1 in cancer cells *via* JQ1, polarizing macrophages to M1, suppressing regulatory T cell (Treg) infiltration, enhancing CD8^+^ T cell presence and activity at the tumor site using mouse models (Wang et al., 2022). Other EV and nanoparticle-based approaches to modulating the immune system have been reviewed elsewhere (Chen et al., 2021).

### Clinical applications of EVs

As of writing, there are several clinical trials involving the use of EVs that are active or recruiting. These trials are employing EVs as drug carriers and as biomarkers for cancer prediction, stage, prognosis, diagnosis, and to determine tumor response to therapeutic treatment (Table 4). Gargiulo et al. identified a set of EV-related genes that were found to be correlated with worse outcomes in patients with chronic lymphocytic leukemia, validating the potential use of EVs as biomarkers for predicting disease progression and patient prognosis (Gargiulo et al., 2022). Circulating cancer-derived EVs have potential utility as biomarkers in liquid biopsies (Yu et al., 2021). In contrast to a tissue biopsy which involves an invasive procedure and does not provide real-time information, liquid biopsies are minimally invasive and include EVs, circulating tumor DNA/RNA/protein, and tumor educated platelets and other circulating components (Liu J. et al., 2021; Li D. et al., 2021). EVs from liquid biopsies, therefore, have exciting potential as cancer biomarkers. Recent studies have focused on the utility of characterizing EVs and their cargo as a predictive biomarker for diseases such as cancer (Urabe et al., 2019), cardiovascular disease (Lennon et al., 2022), diabetes (Kong et al., 2019; Liu C. et al., 2021), and diabetic complications (Jia et al., 2016; Li et al., 2018). However, in regards to using EVs in therapeutics, there remains several concerns such as their stability, dosing strategy, off-target effects, and feasibility in terms of production, expansion, and application.

**TABLE 4:** Current EV-related clinical trials. Information is current as of 26 January 2023 and was found through clinicaltrials.gov by searching for clinical trials involving extracellular vesicles or exosomes.

Use	Disease	Cargo or target (if applicable)	Status	NCT identifier	Citation
Carrier and maintenance immunotherapy	Non-small cell lung cancer	Tumor antigens	Completed	NCT01159288	Besse et al. (2016)
Carrier	Pancreatic cancer	siRNA against KrasG12D	Recruiting	NCT03608631	Kamerkar et al. (2017)
Carrier	Hepatocellular carcinoma, gastric cancer liver metastases, and colorectal cancer	Anti-sense oligonucleotide targeting STAT6	Recruiting	NCT05375604	Kamerkar et al. (2022)
Biomarker	Lung metastases from osteosarcoma	RNA cargo characterization	Unknown	NCT03108677	Bao et al. (2018)
Biomarker	Lung cancer	-	Active	NCT04529915	-
Biomarker	Pancreatic cancer	-	Recruiting	NCT02393703	-
Biomarker	Lung cancer	-	Recruiting	NCT04629079	-
Biomarker	Gastric cancer	LncRNA-GC1	Recruiting	NCT05397548	Xin Guo et al. (2020)
Biomarker	Thyroid cancer	Thyroglobulin and Galectin-3	Recruiting	NCT04948437	-
Biomarker	Non-small cell lung cancer	-	Recruiting	NCT05424029	-
Biomarker	Lung cancer	-	Recruiting	NCT04939324	-
Predict response to anlotinib	Non-small cell lung cancer	-	Not yet recruiting	NCT05218759	-
Monitor disease	Sarcoma	RNA cargo characterization	Recruiting	NCT03800121	-
Immune evasion mechanism	Diffuse large B-cell lymphoma	Cargo characterization	Recruiting	NCT03985696	-
Screening tool	Oropharyngeal squamous cell carcinoma	-	Recruiting	NCT02147418	-
Immune evasion mechanism	Liver cancer	Cargo characterization	Recruiting	NCT05575622	-
Biomarker	Pancreatic adenocarcinoma	-	Recruiting	NCT03334708	-
Biomarker for disease progression	Thyroid cancer	-	Not yet recruiting	NCT05463107	-
Early detection	Breast cancer	miRNA characterization	Not yet recruiting	NCT05417048	-
Biomarkers for predicting treatment response	Small cell lung cancer	Long RNA characterization	Recruiting	NCT05191849	-

### Limitations of EV-based therapy

Although there is potential utility of EVs in treating disease, there are limitations and caveats of an EV-based treatment approach. EV size influences tumor uptake; comparison of EVs 50–200 nm containing camptothecin revealed that 50 nm EV penetrated tumors at a higher rate, leading to increased efficacy (Tang et al., 2013). As mentioned above, EPR is a key feature of tumors that enhances nanomedicine accumulation and localization of tumors compared to normal tissues. However, EPR does not show the same characteristics across all tumors, complicating the prediction of tumor nanoparticle uptake (Prabhakar et al., 2013). Furthermore, only a small number of nanoparticles will be delivered to the tumor. Therefore, it is important to determine how to promote active homing and targeting of nanoparticles to tumors, rather than relying on passive targeting (Clemons et al., 2018). A multivariate analysis conducted by Wilhelm et al. surveying the literature from 2005 to 2015 identified an average delivery efficiency of 1.48% of the administered nanoparticle dose (Wilhelm et al., 2016). A later study conducted by Cheng et al. utilized a pharmacokinetic modelling approach to analyze data from 2005 to 2018 and found an average delivery efficiency of 2.24% at 24 h, but 1.23% at 168 h after intravenous administration of the nanoparticle therapy (Cheng et al., 2020). Interestingly, EVs shed from CAR-T cells have been found to retain the CAR on their surface and maintain potent anti-tumor effects *in vitro* and *in vivo* (Fu et al., 2019)*.* CAR-T cell membranes coated with nanoparticles resulted in enhanced anti-tumor abilities *in vitro* and *in vivo* in a liver cancer model (Ma et al., 2020). Because of low nanoparticle delivery efficiency, there remains several concerns regarding the bioavailability, biodistribution, cost, and safety profiles of administering nanoparticles in large doses to achieve sufficient tumor delivery to overcome these roadblocks. However, there are many promising ongoing clinical trials geared towards addressing these concerns and optimizing treatment conditions in patients with different kinds of cancer (Anselmo and Mitragotri, 2019).

## Conclusion

The past decade has witnessed a significant increase in research surrounding EVs and their potential clinical applications. Here, we discussed EV formation and their diverse and heterogenous DNA, RNA, and protein cargo. We also reviewed implications for EVs in cancer progression, particularly within the context of the TME, and how they may facilitate intra- and inter-cellular communication to promote metastasis and further growth. Studies involving EVs have expanded in recent years with many showing considerable promise *in vitro, in vivo*, and in clinical trials. However, significant challenges remain in identifying and implementing cost and time efficient methods for EV isolation to maximize their yield, integrity, and stability. Furthermore, specific methods are needed to characterize EVs and their cargo, particularly in differentiating them from non-EV material. This is essential for accurate characterization of EVs as predictive biomarkers of disease and elucidating their physiological role.

As the EV field has expanded, numerous questions have emerged including the diversity and complexity of EV cargo and the role of this cargo in cancer onset and progression. A relatively unexplored area is the packaging of reactive molecules and their by-products in EVs. An intriguing example of this is methylglyoxal (MG) and methylglyoxal-derived advanced glycation end products (MG-AGEs), most recently reviewed here (Lai et al., 2022). MG-AGEs are correlated with numerous disease states, but their role in causation is not clear. They are proposed to induce inflammation and act as signaling molecules by activating their receptor, RAGE, a cascade that is correlated with many different disease pathologies. The presence and role of both MG and MG-AGEs in EVs have not yet been characterized. However, given their correlation with disease, it is conceivable that their presence in EVs may participate in disease onset and progression. Therefore, it is of interest to determine if and how EVs may serve a mechanistic role in shuttling these and other reactive molecules between cells, potentially serving as a way of propagating cellular signals and stress. There is also a gap in knowledge in the impact of intra-EV protein post-translational modifications, DNA and RNA adducts, and reactive oxygen species on EV stability and abundance. Additionally, there is interest in elucidating differences in the levels of these molecular changes in EVs derived from various cell types to use as biomarkers to determine from which cells EVs originate. Further characterization of EV membrane protein modifications and diversity also has potential application to help determine the source of specific EVs and as an additional tool for EV enrichment.

The widespread abundance, biological nature, and physical characteristics of EVs make them attractive for clinical use as therapeutic vesicles, targets for treatment, and biomarkers. As the field continues to expand and the number of clinical trials investigating EVs increases, it is likely EVs will be implemented for clinical use. Continued refinement and improvement of isolation and characterization techniques will be instrumental in the successful implementation of EVs in clinical applications.

## Glossary

ApoBDs: apoptotic bodies
AR: androgen receptor
ARF6: ADP-ribosylation factor 6
BBB: blood-brain barrier
CAR-T: chimeric antigen receptor re-directed T cells
CEdG: R,S-N2-carboxyethyl-deoxyguanosine
CEG: R,S-N2-carboxyethyl-guanosine
CEL: carboxyethyllysine
CSF-1: colony stimulating factor-1
dUC: differential ultracentrifugation
ECM: extracellular matrix
EGFR: epidermal growth factor receptor
EMT: epithelial-mesenchymal transition
EPR: enhanced permeability and retention effect
ER: endoplasmic reticulum
ESCRT: endosomal sorting complex required for transport
EVs: extracellular vesicles
IFN-γ: interferon gamma
IL-1β: interleukin-1β
IL-6: interleukin-6
ISEV: International Society for Extracellular Vesicles
ITGβ3: integrin-β3
ITGβL1: integrin beta-like 1
lncRNAs: long-coding RNAs
MDSCs: myeloid-derived suppressor cells
MISEV: Minimal Information for Studies of Extracellular Vesicles
MG: methylglyoxal
MG-AGEs: methylglyoxal-derived advanced glycation end products
MHC: major histocompatibility complex
miRNA or miR: micro-RNA
MLCK: myosin light chain kinase
MVs: microvesicles
NTA: nanoparticle tracking analysis
NDPK: nucleoside diphosphate kinase A and B
NFκB: nuclear factor kappa-B
PANX 1: pannexin 1
PCa: prostate cancer
PD-1: programmed cell death protein 1
PD-L1: programmed death ligand 1
PLD2: phospholipase D2
PEG: polyethylene glycol
PMN: pre-metastatic niche
RAGE: receptor for advanced glycation end products
PRR RIG-I: pattern recognition receptor retinoic acid-inducible gene I
RANKL: receptor activator of nuclear factor kappa-B ligand
RBP: RNA-binding protein
RNAi: RNA interference
ROCK: Rho-associated protein kinase
ROS: reactive oxygen species
SEC: size exclusion chromatography
RN7SL1: Recognition Particle 7SL1
SCARB1: scavenger-receptor type B-1
sEV: small extracellular vesicles
SIPA 1: signal-induced proliferation-associated 1
SMase: sphingomyelinase
SNARE: N-ethylmaleimide-sensitive factor attachment protein receptor
TGF-β: transforming growth factor β
TME: tumor microenvironment
TNBC: triple negative breast cancer
TNFα: tumor necrosis factor alpha
Treg: regulatory T cell
tRNA: transfer RNA
VEGF: vascular endothelial growth factor
